# “Cost-effectiveness of ACL treatment is dependent on age and activity level: a systematic review”

**DOI:** 10.1007/s00167-022-07087-z

**Published:** 2022-08-23

**Authors:** R. Deviandri, H. C. van der Veen, A. M. T. Lubis, I. van den Akker-Scheek, M. J. Postma

**Affiliations:** 1 Department of Orthopedics, University of Groningen, University Medical Center Groningen, Hanzeplein 1, 9713 GZ Groningen, The Netherlands; 2 Department of Physiology, Faculty of Medicine, Universitas Riau, Pekanbaru, Indonesia; 3 Division of Orthopedics, Arifin Achmad Hospital, Pekanbaru, Indonesia; 4 Department of Orthopedics-Faculty of Medicine, Universitas Indonesia/Cipto Mangunkusumo Hospital, Jakarta, Indonesia; 5 Department of Health Sciences, University of Groningen, University Medical Center Groningen, Groningen, The Netherlands; 6 Department of Economics, Econometrics & Finance, Faculty of Economics & Business, University of Groningen, Groningen, The Netherlands; 7 Department of Pharmacology & Therapy, Universitas Airlangga, Surabaya, Indonesia; 8 Center of Excellence in Higher Education for Pharmaceutical Care Innovation, Universitas Padjadjaran, Bandung, Indonesia

**Keywords:** Anterior cruciate ligament, Surgery, Rehabilitation, Physiotherapy, Health economics

## Abstract

**Purpose:**

To systematically review the literature on health-economic evaluations of anterior cruciate ligament (ACL) injury between reconstruction surgery (ACLR) and non-operative treatment (NO) and suggest the most cost-effective strategy between the two.

**Methods:**

All economic studies related to ACLR versus NO post-ACL injury, either trial based or model based, published until April 2022, were identified using PubMed and Embase. The methodology of the health-economic analysis for each included study was categorized according to the four approaches: cost-minimization analysis (CMA), cost-effectiveness analysis (CEA), cost–benefit analysis (CBA), and cost-utility analysis (CUA). The quality of each included study was assessed using the Consensus on Health Economic Criteria (CHEC) list.

**Results:**

Of the seven included studies, two compared the strategies of early ACLR and NO alone, and five compared early ACLR and NO with optional delayed ACLR. All studies performed a CUA, and one study performed a CBA additionally. The CHEC scores of the included studies can be considered good, ranging from 15 to 18 from a maximum of 19. Applying the common standard threshold of $50,000 per QALY, six studies in young people with high-activity levels or athletes showed that early ACLR would be preferred over either NO alone or delayed ACLR. Of six studies, two even showed early ACLR to be the dominant strategy over either NO alone or delayed ACLR, with per-patient cost savings of $5,164 and $1,803 and incremental per-patient QALY gains of 0.18 and 0.28, respectively. The one study in the middle-aged people with a moderate activity level showed that early ACLR was not more cost-effective than delayed ACLR, with ICER $101,939/QALY using the societal perspective and ICER $63,188/QALY using the healthcare system perspective.

**Conclusion:**

Early ACLR is likely the more cost-effective strategy for ACL injury cases in athletes and young populations with high-activity levels. On the other hand, non-operative treatment with optional delayed ACLR may be the more cost-effective strategy in the middle age population with moderate activity levels.

**Level of evidence:**

Systematic review of level III studies.

**Supplementary Information:**

The online version contains supplementary material available at 10.1007/s00167-022-07087-z.

## Introduction

Anterior cruciate ligament (ACL) injury is a common sports injury with an overall age- and sex-adjusted annual incidence of 74.6 per 100,000 person-years [33]. ACL reconstructive surgery (ACLR) is frequently performed for managing ACL injuries by orthopedic surgeons, although in selected patients ACL injuries can be managed successfully with non-operative treatment with rehabilitation (NO) [4, 16, 36, 37]. One of the most important reasons for surgery is to regain a stable knee and return to sports as quickly as possible, particularly for young and active people [4, 23, 38].

Considerable debate remains regarding the preferred strategy for managing individuals following an ACL injury. In their systematic review, Smith et al. found no statistically or clinically relevant differences in clinical outcomes between ACLR and NO [37]. Yet, as costs may differ, it is relevant to also look at this debate from a health-economic perspective. Such a review could be very helpful in further elucidating the preference for strategies, potentially within specific populations. Insight into the economic aspects could support the development of recommendations for clinical practice guidelines, explicitly taking into account health-economic evidence such as costs and cost-effectiveness [40].

To our knowledge, no systematic review has yet been conducted on the available literature on ACL treatment strategies to directly compare ACLR versus NO from a health-economic perspective. The purpose of this study was to systematically review the literature, including both trial based and model-based health-economic evaluations of ACL injury between ACLR and NO, and suggest the most cost-effective strategy between the two.

It was hypothesized that reconstructive surgery is more cost-effective in athletes and young populations, while non-operative treatment with optional delayed reconstructive is a more cost-effective strategy in the middle-aged population.

## Materials and methods

This review was registered in PROSPERO under registration no. CRD42020206124. This review followed the PRISMA guidelines [25].

### Search strategy

The primary search was performed using PubMed and Embase [40], for the timeframe 1966 to March 2022. Search terms or phrases represented in Medical Subject Headings (MeSH) were used for PubMed. Subsequently, the terms or phrases used in PubMed were translated to the Embase database. To refine the results, a search strategy using the Boolean operator “OR” within sequences of terms with close or similar meanings and “AND” for one or more sequences of terms that incorporated utterly different meanings was conducted. Whole terms and phrases for either PubMed or Embase were identified by two persons–one is the author (RD), the other a librarian at our institution (KIS)–who dealt with the search strategy. Lastly, the reference lists of each included study and review paper on this topic were assessed, and snowballing was subsequently performed if new references came up. The key strings for the PubMed search strategy are shown in Table 1, including the underlying PICO (patients, intervention, comparator, outcomes).

**Table 1:** PubMed search strategy

P: anterior cruciate ligament injury
I: anterior cruciate ligament reconstruction
C: rehabilitation
O: cost analysis
(“Anterior cruciate ligament”[Mesh] OR “anterior cruciate ligament/injuries” [Mesh] OR ACL[tiab] OR Anterior Cruciate Ligament*[tiab])
AND
(“Anterior cruciate ligament reconstruction” [Mesh] OR “reconstructive surgical procedures”[Mesh] OR reconstruct*[tiab] OR surg*[tiab] OR “surgery” [Subheading] OR operat*[tiab])
AND
(“Physical and rehabilitation medicine” [Mesh] OR “rehabilitation” [Subheading] OR “Orthopedic Procedures”[Mesh] OR rehabil*[tiab] OR “physical therapy modalities”[Mesh] OR physical therap*[tiab] OR nonoperat*[tiab] OR conservative[tiab])
AND
(“Costs and cost analysis” [Mesh] OR cost*[tiab] OR economic*[tiab] OR socioeconomic*[tiab])

### Eligibility criteria

Studies were deemed eligible if they evaluated the clinical benefits and costs related to ACLR versus NO after ACL injury and were either trial based or model based. Both child and adult populations were included, although they would be analyzed separately. All studies were included irrespective of publication language and year. Studies issued as commentary, editorials, research protocols, and reviews were excluded. The process of literature searches, including screening of titles and abstracts, the data extraction, and subsequently the quality assessment of papers using the Consensus on Health Economic Criteria (CHEC), was performed independently by two authors (RD and HCvdV). Any disagreement between the two authors was resolved through discussion. If consensus could not be reached, this was resolved by the third reviewer (MJP).

### Identification of eligible studies

Titles and abstracts of each identified citation were reviewed comprehensively. The full text of each potentially eligible paper was subsequently reviewed. If the full text of the paper satisfied the eligibility criteria, it was included in the final review.

### Data extraction

Extracted data included the fields of authorship, year of publication, journal, country, sample size, sex, direct and indirect costs, utilities’ values, time horizon, discount rates, outcome, transition probabilities of the outcome, and results of sensitivity analysis.

### Cost analysis and data synthesis

The methodology of the health-economic analysis and outcomes for each included study were categorized according to four approaches [6]: (1) cost-minimization analysis (CMA), where the costs of each alternative are analyzed straightforwardly in terms of monetary costs, assuming equal health outcomes for each intervention; (2) cost-effectiveness analysis (CEA), where the outcomes are expressed in a natural unit of health such as number of patients with clinical improvement, cures, and life-years gained; (3) cost–benefit analysis (CBA), where each intervention is analyzed in terms of benefit and cost, with all aspects expressed in monetary units; and (4) cost-utility analysis (CUA), often as the preferred technique in which each intervention is analyzed in terms of cost and utilities indicating preferences for health outcomes, synthesized in cost per quality-adjusted life-year (QALY). For types of economic evaluations, cost perspectives with corresponding potential components of (1) direct costs such as those for reconstructive procedure, hospitalization, radiology, laboratory, and drugs, and (2) indirect costs reflecting productivity losses were taken into account.

Costs were made comparable among individual studies using currency conversions to US$ and correcting for inflation rates. Inflation rates were calculated based on the 2020 annual Gross Domestic Product Deflator (GDPD) index from the International Monetary Fund (IMF) World Economic Outlook Database “GDP Deflator Index” dataset for each respective country [35]. The free web-based tool developed by the Campbell and Cochrane Economics Methods Group (CCEMG) was used to adjust all of the costs as suggested by van Mastrigt et al. [41]. If the author did not state the actual year of the data cost, the price level was assumed as being the same as the year when the study was conducted.

The activity levels were classified based on Tegner scales from 0 to 10. The values < 2, 2–4, 5–7, 8–9, and 10 are categorized as very low, low, moderate, high, and very high-activity level, respectively. Hence, the ages group were classified into five; < 18, 18–30, 31–45, 45–65, and > 65 as pediatric, young people, middle age, old adult, and elderly, respectively. Further, an early ACLR was defined as ACLR treatment within 12 weeks of injury, while delayed ACLR was defined as ACLR treatment more than 12 weeks after injury [8, 13, 29].

Due to heterogeneity and diversity of all included data, a meta-analysis might not possible. Therefore, a narrative data synthesis would be performed by presenting all findings in summative form, including table and figures [26].

### Critical appraisal

Each included study was appraised using the CHEC list to assess the quality of reporting health-economic outcomes, including potential bias in individual studies. The CHEC is a validated and reliable appraisal tool for evaluating health-economic studies, either model based or trial based [10, 41]. This CHEC instrument comprises a 19-item list on study design (4 items), time horizon, actual perspective, cost evaluation (5 items), outcome measurements (3 items), discounting, conclusion, generalization, conflict of interest, and ethical issues [10, 11]. These items can be conceived to reflect the minimum requirements for health-economic papers. As the CHEC does not specify summary scores, the classification and the score limits were defined by the authors. One point was scored for “yes”, indicating a satisfactorily addressed item. Marks of “unclear” and “no” were scored half a point and zero, respectively [27]. Hence the minimum and maximum scores for the individual studies ranged from 0 to 19. The values < 10, 10–14.5, and > 14.5 are indicative of low, moderate, and high-quality economic evaluation, respectively.

## Results

### Search results

**Fig. 1:**
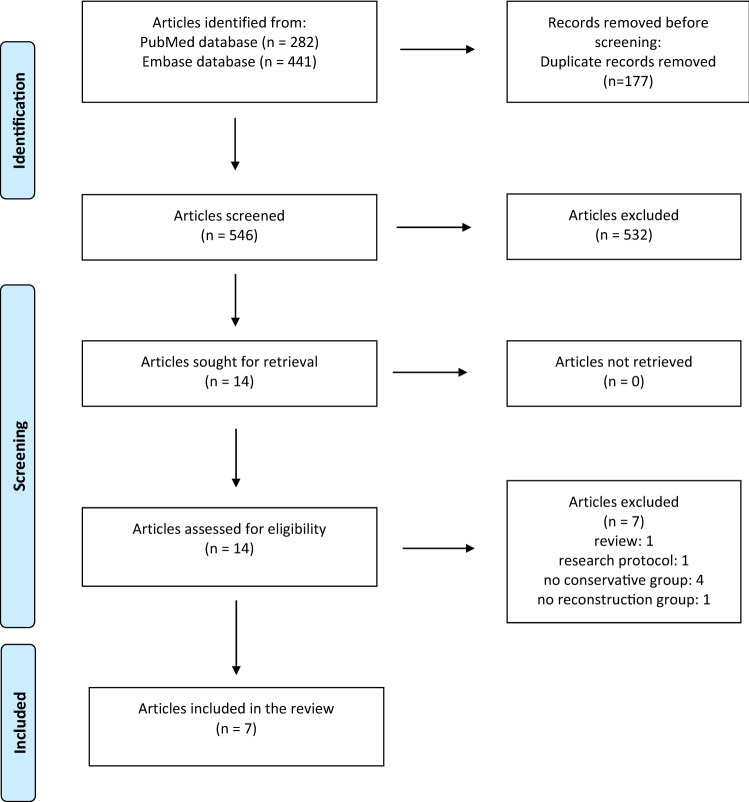
PRISMA flow diagram [20]

This review initially identified a total of 282 and 441 articles from the PubMed and Embase databases, respectively. After removing duplicates (177), the remaining 546 titles and abstracts were screened. Based on the predefined inclusion and exclusion criteria, a total of 532 articles were excluded. After the full-text screening of the remaining 14 articles, a total of 7 articles were included in this systematic review. Figure 1 shows a flow diagram of the selection.

### General characteristics of included studies

The general characteristics of the included studies are presented in Table 2. Of the seven included studies, four originated in the USA and the other three in Europe. The reviewed studies concerned two trial-based studies based on randomized controlled trials (RCTs) [8, 19] and five model-based studies [12, 14, 21, 22, 38]. All studies performed a CUA, one study performed a CBA additionally [19], and no CMAs and CEAs were identified. There were five studies on young people with high-activity levels [12, 14, 19, 21, 21], one study particularly addressing a group of athletes [38], and another generally involving young to middle-aged persons with a moderate activity level [8].

**Table 2:** General characteristics of included studies

References	Country	Studies’ specificities					Type of economic evaluation	Outcome measure	Study design	Time horizon
		Gender	Age	Early ACLR	CTa/delayed ACLR	Group				
Gottlob et al. [12]	USA	M, F	21–27	363	307	Athletes and young people	CUA	QALYs, cost	Model-based	7 years
Farshad et al. [10]	CH	M, F	27.5	229	155	Young people	CUA	QALYs, cost	Model-based	7.5 years
Mather et al. [18]	USA	M, F	26 ± 11	988* + 62**	59	Young people	CUA	QALYs, cost	Model-based	6 years & lifetime
Mather et al. [17]	USA	M, F	26 ± 11	807* + 62**	59	Young people	CUA	QALYs, cost	Model-based	6 years
Kiadaliri et al. [15]	SE	M, F	18–35	61	59	Young people	CUA& CBA	QALYs, cost	Trial based	5 years
Stewart et al. [38]	USA	M, F	Not reported	2719	147	Athletes	CUA	QALYs, cost	Model-based	6 years
Eggerding et al. [8]	NL	M, F	18–65	85	82	young to middle-aged people	CUA	QALYs, cost	Trial based	2 years

*CH* Switzerland, *SE* Sweden, *NL* The Netherlands, *QALY* quality-adjusted life-year, *CUA* cost-utility analysis, *CBA* cost–benefit analysis, *early ACLR* early anterior cruciate ligament reconstruction surgery, *CTa* conservative treatment alone, *delayed ACLR* conservative treatment with optional delayed anterior cruciate ligament reconstruction

^*^MOON database, **KANON study

Of the seven included studies, two compared the strategy between early ACLR and NO alone [14, 22], and five compared between early ACLR and non-operative treatment with optional delayed ACL reconstructive (delayed ACLR) [8, 12, 19, 21, 38]. Three studies performed a CUA from a societal perspective [19, 22, 38] and three others analyzed the data from a healthcare system’s perspective [12, 14, 21]. Another study performed a CUA from both perspectives [8]. Costs and value of QALYs in included studies were discounted at 3% annually, except for one study that set the discount rates at 4% for cost and 1.5% for QALYs [8]. One study did not explain the discount rates used [12]. Using the common standard threshold of $50,000 per QALY, six studies showed that early ACLR was more cost-effective than either NO alone or delayed ACLR [12, 14, 19, 21, 22, 38]. In particular, one study showed that early ACLR was more cost-effective than NO alone with an incremental cost-effectiveness ratio (ICER) of $9023/QALY [14], and three studies showed that early ACLR was more cost-effective than delayed ACLR with ICERs of 3461/QALY, $43,152/QALY, and $24,847/QALY respectively [12, 19, 38]. Two additional studies estimated that early ACLR would be the dominant strategy over either NO alone or delayed ACLR in the short-to-medium term with per-patient cost savings of $1803 and $5164, and incremental per-patient QALYs gains of 0.28 QALY and 0.18 QALY, respectively [21, 22]. One study showed that early ACLR was not cost-effective compared to delayed ACLR, with an ICER of $101,939/QALY using the societal perspective and $63,188/QALY using the healthcare system perspective [8]. The results of the included studies are presented in Table 3.

**Table 3:** Results of included studies regarding index year, perspective, adjusted costs in US$ at 2020 price levels (using appropriate inflation rates for corrections), effectiveness results, ICER, and discount rate

References	Index year	Perspective	Costs in US$ at 2020 price levels	Effectiveness	ICER	Discount rate per year
Gottlob et al. [12]	1997	Healthcare	Total cost early ACLR: $18,130 Total cost CTa: $3594	Early ACLR more effective than CTa gaining 1.61 QALYs per person	$9023/QALY	Cost and QALYs:3%
Farshad et al. [10]	2010	Healthcare	Total cost early ACLR:$11,352 Total cost delayed ACLR: $10,947	Early ACLR more effective than delayed ACLR gaining 0.12 QALYs per person	$3461/QALY	Not reported
Mather et al. [18]	2012	Societal	Total cost early ACLR for long term: $43,721 Total cost CTa for long term: $101,545	Short-to-medium-term: early ACLR more effective than CTa gaining 0.18 QALYs per person Long-term: early ACLR more effective than CTa gaining 0.72 QALYs per person	Early ACLR is dominant (per-patient cost savings $5164) early ACLR is dominant (per-patient cost savings $57,824	Cost and QALYs:3%
Mather et al. [17]	2012	Healthcare	Total cost early ACLR: $22,804 Total cost delayed ACLR: $24,606	Early ACLR more effective than delayed ACLR gaining 0.28 QALYs per person	Early ACLR is dominant (per-patient cost savings $1803)	Cost and QALYs:3%
Kiadaliri et al. [15]	2011	Societal	Total cost early ACLR: $31,240 Total cost delayed ACLR: $25,630	Early ACLR more effective than delayed ACLR gaining 0.13 QALYs per person	$43,152/QALY	Cost and QALYs:3%
Stewart et al. [38]	2015	Societal	Total cost early ACLR: $22,216 Total cost delayed ACLR: $12,973	Early ACLR more effective than delayed ACLR gaining 0.35 QALYs per person	$24,847/QALY	Cost and QALYs:3%
Eggerding et al. [8]	2018	Societal and healthcare	Using societal perspective: Total cost early ACLR: $19,495 Total cost delayed ACLR: $15,071	Early ACLR more effective than delayed ACLR gaining 0.043 QALYs per person	Using societal perspective: $101,939/QALYs	Cost: 4% QALYs:1.5%
			Using healthcare perspective: Total cost early ACLR: $8,302 Total cost delayed ACLR: $5,564		Using healthcare perspective: $63,188/QALYs	

*ICER* incremental cost-effectiveness ratio, *QALY* quality-adjusted life-year, *early ACLR* early anterior cruciate ligament reconstruction surgery, *CTa* conservative treatment alone, *delayed ACLR* conservative treatment with optional delayed anterior cruciate ligament reconstruction

Considering the modeling types, Mather et al. [21, 22] applied a Markov model on both the Knee Anterior Cruciate Ligament, Non-Surgical versus Surgical Treatment (KANON) study and the Multicenter Orthopedic Outcomes Network (MOON) databases. Stewart et al. [38] used a Markov model based on a literature review. The other studies used decision tree models based on a literature review [12, 14], and two studies based their analysis on RCTs [8, 19]. The value’s score, source of data, and modeling strategy of each study are presented in Table 4.

**Table 4:** Analysis of uncertainty

References	Scenarios & Sensitivity analyses applied	Sensitivity analysis	
		Parameters to which results were most sensitive	Results
Gottlob et al. [12]	Three best–worst-case scenarios: (1) utility value range from athlete (best-case) to non-athlete (worst-case); (2) results from best surgical and worst non-operative vs results from worst surgical and best non-operative; (3) most favorable utilities (athlete) and outcome vs least favorable utilities (non-athlete) and outcome	Cost, utilities, and outcome	early ACLR preferred strategy over CTa for all scenarios, ICER early ACLR for athletes and non-athletes, range $7,805 to $10,974/QALY
Farshad et al. [10]	Monte Carlo simulation using 10,000 samples	Sequelae of ACL injury: meniscus injury, osteoarthritis	early ACLR preferred strategy over delayed ACLR
Mather et al. [18]	Monte Carlo simulation using 10,000 samples 1-way sensitivity analysis	Rate of instability after initial conservative treatment (if ICER as outcome) Age (if cost only as outcome)	In 93% of cases, early ACLR preferred at WTP threshold of $50,000/QALY (short-to-medium-term) In 59% of cases, early ACLR preferred at WTP threshold of $50,000/QALY (long term)
Mather et al. [17]	Monte Carlo simulation used 10,000 samples 1-2-and 3-way sensitivity analysis	Rate of instability after initial conservative treatment (if ICER as outcome) Rehabilitation visit ratio, no. patients choosing delayed reconstruction, cost of early ACLR (if cost only as outcome)	In 78% of cases, early ACLR preferred at WTP threshold of $50,000/QALY
Kiadaliri et al. [15]	1-way and multi-way sensitivity analysis Bootstrapping technique used 10,000 samples	Cost of secondary procedure, productivity losses, second ACL reconstruction, operation not related to the knee, and outliers cost and QALY	In 66% of cases, early ACLR preferred at WTP threshold of SEK500,000/QALY
Stewart et al. [38]	Monte Carlo simulation used 10,000 samples 1 and 2-way sensitivity analysis	QoL of returning or not returning to play, cost, and duration of follow-up (if ICER as outcome)	In 80% of cases, early ACLR preferred at WTP threshold of $50,000/QALY
Eggerding et al. [8]	Bootstrapping technique used 5000 samples	NA	In 35% of cases, early ACLR preferred at WTP threshold of €50,000/QALY (using societal perspective) In 54% of cases, early ACLR preferred at WTP threshold of €50,000/QALY (using healthcare perspective)

*ICER* incremental cost-effectiveness ratio, *QALY* quality-adjusted life-year, *early ACLR* early anterior cruciate ligament reconstruction, *CTa* conservative treatment alone, *delayed ACLR* conservative treatment with optional delayed anterior cruciate ligament reconstruction, *QoL* quality of life, *WTP* willingness to pay, *NA* not applicable

All studies performed a sensitivity analysis or analysis of uncertainty for all critical variables whose values were uncertain. Mather et al. [21, 22] explained that ICER was sensitive to the rate of knee instability after an initial non-operative treatment in the model. Farshad et al. [12] explained that ICER was sensitive to the sequelae of ACL injury such as meniscal lesion and osteoarthritis. Stewart et al. [38] showed that ICER was most sensitive to the exact value reflecting quality of life, returning to play or not, costs of early ACLR and delayed ACLR, and duration of follow-up in the model. The analysis of the uncertainty results of the included studies is presented in Table 5.

**Table 5:** Comparisons of transition probabilities, utilities, sources of cost data, and modeling strategy

References	Transition probabilities	Applied health utility	Source of data	Modeling strategy
Gottlob et al. [12]	Risk of late meniscal surgery: early ACLR vs CTa = 0.78:3.5	Vignette method, based on functional activity level, by giving a questionnaire to healthy students Utility range: 0–1 Athlete return vs no return to play: 1.00 vs 0.43–0.62	Cost of early ACLR from database of Chicago hospital Cost of CTa based on local referral facility's prices	Decision tree
Farshad et al. [10]	Revision rate after initial early ACLR: 3.5% Rate of delayed reconstruction after initial CT: 16% Rate of sequelae after early ACLR: 34% (86% OA, 14% meniscal lesion) Rate of sequelae after initial CT: 77% (74% OA, 26% meniscal lesion)	Vignette method, based on functional activity level by expert opinion Utility range: 0–1 Early ACLR: 0.78 CT: 0.66	Orthopedic University Hospital Balgrist, Switzerland Cost of early ACLR and delayed ACLR: Swiss National Insurance System for Injuries (UVG)	Decision tree
Mather et al. [18]	Rate of reoperation after initial early ACLR: 36% Rate of reoperation after initial CT: 26% Rate of OA: 3% Annual re-injury after early ACLR: 1.5% Rehabilitation ratio CTa/early ACLR: 1	SF6D Unstable knee: 0.71 Stable knee: 0.82	MOON database and KANON study Cost of early ACLR and CTa: Medicare insurance	Markov Model
Mather et al. [17]	Rate of delayed reconstruction after initial CT in 2 years: 55% Rate of reoperation after initial early ACLR: 32% Rate of reoperation after initial CT: 36% Rehabilitation ratio delayed ACLR/early ACLR: 1.125	SF6D Unstable knee: 0.71 Stable knee: 0.82	MOON database and KANON study Cost of early ACLR and delayed ACLR: Medicare insurance	Markov Model
Kiadaliri et al. [15]	Annual re-injury after early ACLR: 2.3% Meniscus operation rate of early ACLR vs. delayed ACLR: 0.8 Rate of delayed reconstruction after initial CT: 51% Rehabilitation ratio delayed ACLR/early ACLR: 0.98	SF6D Early ACLR: 0.61–0.85 Delayed ACLR: 0.61–0.84	KANON study Cost of early ACLR and delayed ACLR: Skane Healthcare	Trial based
Stewart et al. [38]	Probability of return to play: early ACLR vs delayed ACLR: 0.610:0.177 Rate of late cartilage surgery early ACLR vs delayed ACLR; return or no return to play: 0.018/ 0.005 vs 0.043/ 0.022	Convert from SF-36 to EQ5D Athlete return vs not return to play: 0.89–1.00 vs 0.62–0.89	Cost of early ACLR and delayed ACLR: Based on Academic medical center, US-cost accounting system	Markov Model
Eggerding et al. [8]	Rate of delayed reconstruction after initial CT: 50%	EQ5D Early ACLR and delayed ACLR: 0.72–0.84	COMPARE study	Trial based

*early ACLR* early anterior cruciate ligament reconstruction surgery, *CT* conservative treatment, *CTa* conservative treatment alone, *delayed ACLR* conservative treatment with optional delayed anterior cruciate ligament reconstruction, *OA* osteoarthritis; SF6D, Short Form-6 Dimension, *EQ5D* Euro Quality Of Life-5 Dimension, *SF-36* 36-Item Short Form Survey, *MOON* Multicenter Orthopedic Outcomes Network, *KANON* Knee Anterior Cruciate Ligament Nonsurgical Versus Surgical Treatment, *COMPARE* Conservative Versus Operative Methods For Patients With Anterior Cruciate Ligament Rupture Evaluation

### Quality assessment of the included studies

The CHEC scores of the included studies ranged from 15 to 18, indicating the high quality of the economic evaluation. The complete quality assessment of each study is reported in the appendix, supplementary data Table A.1.

## Discussion

The most important finding of this study was that early ACLR is likely a cost-effective strategy for managing ACL injuries in athletes and young populations with high-activity levels. By contrast, for patients in the middle-aged population with moderate activity levels, early ACLR seems an unlikely cost-effective strategy, although this conclusion is based on only one study.

Our findings for athletes and young populations are in line with those of a previous review that described early ACLR independently as a more cost-effective strategy than NO [1, 24, 32]. All studies in the previous review were performed in young populations with high-activity levels. It was found that in athletes and young populations with high-activity levels—using the common standard threshold of $50,000 per QALY—early ACLR was more cost-effective than either NO alone or delayed ACLR [12, 14, 19, 21, 22, 38]. In our review, also it was included a study among the general population (patients with moderate activity levels, aged 18–65, mean aged 31 ± 10). On the other hand, the study among the more general population revealed that early ACLR was not a cost-effective strategy after ACL injury compared to delayed ACLR, with an ICER of $101,939 using the societal perspective and an ICER of $63,188 using the healthcare perspective [8]. From these results, it could be concluded that patients’ age and physical activity level are substantial for determining the most cost-effective strategy in ACL injury cases. One of the important reasons for this finding may be due to the greater need to regain a stable knee and return to sports as quickly as possible, particularly for young and active people with high-activity level [4, 16, 23]. Therefore, the clinician should pay more attention to the patients’ age and physical activity level of the patients when considering the appropriate treatment for the ACL injuries cases.

There is increasing evidence for a positive correlation between physical activity level and health state utility value [6, 18, 20], making it clear that the health state utility is an important component to determine the most cost-effective strategy [30, 31, 40, 41]. Young and active people who have high health state utility and who highly value returning to their previous activity level but are unable to do so, would have a low ICER for the early ACLR strategy. Hence the more important returning to previous level activity is, the more cost-effective early ACLR becomes [38].

Various methods could be used to measure health state utility values [31]. In our review, two studies determined it by the vignette valuation method [12, 14]. This method is preceded by determining the vignette health state a priori, then each vignette state can be valued either by clinical experts or a specific population. The valuation of vignettes also can be performed using preference elicitation techniques such as standard gambler (SG) or time trade-off (TTO). Using this method, Gottlob et al. [14] showed utility values of 1.0 for patients who could return to their previous activity level after treatment and 0.43–0.62 for patients who could not. This wide range of utility values for ACL injury patients who received treatment resulted in a more cost-effective early ACLR strategy than NO alone thanks to higher incremental effectiveness and lower ICER. Other studies used the preferences-based method to determine utility value with either the Short Form-6 Dimension (SF-6D) or the Euro Quality of life-5 Dimension (EQ-5D) [8, 19, 21, 22, 38]. Stewart et al. [38] showed utility values of 0.89–1.0 for patients who could return to their previous activity level after treatment and 0.62 to 0.89 for those who could not, using the EQ-5D converted from SF-36 value; the differences they encountered in utility between the two patient groups are lower than the aforementioned value used by Gottlob et al. [14]. So, although still cost-effective, it makes the ICER of the early ACLR strategy higher and thus less attractive. Further, two of the studies used similar primary data sources from the KANON study and determined utility values using SF-6D [19, 21]. Kiadaliri et al. [19] reported a utility value between 0.61 and 0.85 with incremental effectiveness of 0.13 QALYs over 5 years, while Mather et al. [21] reported a utility value between 0.71 and 0.82 with incremental effectiveness of 0.28 QALYs over 6 years. The most recent study used the EQ-5D to determine the health state utility value of the general population of ACL injury patients with a moderate activity level [8]. In that study, patients in both strategies had a utility value between 0.72 and 0.84, with incremental effectiveness of 0.04 QALYs over 2 years. This lower incremental effectiveness between the two strategies yielded early ACLR less cost-effective than the delayed ACLR strategy.

Concerning the NO strategy for ACL injuries, most studies applied delayed ACLR instead of NO alone [8, 12, 19, 21, 38]. However, two studies compared early ACLR with NO alone without an option of crossing over to delayed ACLR if the initial NO failed [14, 22]. The delayed ACLR strategy seems to be more representative of usual care in clinical practice. As it is essential to align economic analysis in common practice and clinical pathways [30], using the delayed ACLR as comparison instead of NO alone is preferred for future research.

A growing body of evidence indicates that people following ACL injury could receive NO interventions before surgery is considered [13, 29, 37, 42]. Eggerding et al. [8] showed that delayed ACLR was more cost-effective than early ACLR in the general population. Obviously, at an early phase, if we were able to adequately recognize those patients who perform well with delayed ACLR from those who do not, this would increase the cost-effectiveness of delayed ACLR as the preferred strategy over early ACLR [6]. On the other hand, if we can identify a pertinent patient for early ACLR treatment and perform this treatment early, it would reduce the cost and make early ACLR a more cost-effective strategy for a specific population. It is supported by Von Essen et al. [9] that early ACLR could reduce socioeconomic costs and is more cost-effective than delayed ACLR in young-active people. Moreover, one should be aware of evidence demonstrating increased risk of meniscus damage with delayed ACLR treatment, especially in young patients [16]. As a consequence, patient selection is an essential part of choosing the appropriate strategy for either early ACLR or delayed ACLR [14, 15].

Subsequently, it is also important to realize that costs alone should not drive decision-making, but rather should costs be taken into consideration during discussion of treatment options with the patient. As economic evaluations are generally based on both cost and effectiveness, a new treatment that is more costly but results in better outcome in comparison with an existing treatment could be considered a more cost-effective and thus preferred strategy. Identification of the patient’s needs is essential before deciding any treatment.

An economic evaluation is performed to determine whether any intervention is more cost-effective than others. Within such studies, outcomes are commonly measured on a utility scale, where 0 is equivalent to death and one equals totally healthy. Such evaluations could use several utility measures, including the SG, TTO, Health Utility Index, EQ-5D, and SF-6D. All claim to measure utility on the same scale. However, as these measures are based on different health descriptions there is growing evidence that they produce different results [2, 3, 17, 39, 43, 44]. Hence agreement on the most appropriate utility measurement method for future cost-effectiveness analyses of treatment options for ACL injury patients is needed, potentially aligned with the preference of Heath Technology Assessment authorities for EQ-5D [3].

In this review there were two high-quality RCTs [8, 19] investigating cost-effectiveness, fulfilling the requirement of comparative effectiveness study from the International Society for Pharmacoeconomics and Outcome Research (ISPOR) guidelines [28]. Results of cost-effectiveness analysis based on RCTs provide direct and substantial evidence for economic assessment [7]. Yet the results of those studies should be interpreted with some caution, as they were conducted over a limited time horizon (often inherent to being RCT-based)—5 years and 2 years, respectively—while the long term is preferred for economic analysis [5, 34]. Plus, the sample sizes of those trials were limited. Sample size specifications are needed to accommodate issues of confounding and treatment heterogeneity. Besides, an RCT cannot answer all policy-relevant questions. Keen focus on identifying the predictors of treatment response heterogeneity needs data sets that are considerably larger than a typical RCT can provide. Although guided basically by RCTs in a narrow population, clinicians, payers, health technology assessment organizations, regulators, and clinical guideline developers are likely to use real-world data studies to sharpen decision-making [5]. It remains the case that a health-economic decision should be interpolated based on heterogeneity rather than data availability from a particular trial. Generally, this decision is extracted using systematic reviews, evidence synthesis, and decision-analytic modeling [34]. It is crucial to choose a good study design, appropriate the cost measurement, value quality of life, and analyze all uncertainty values to convince regulators and other key stakeholders with the results.

This current study has some limitations. Our findings may be not representative of the pediatric and senior populations, as all of the studies in the review included young to middle-aged people. There appears to be a gap in the literature regarding procedures in children and older people.

Further, the results of studies conducted in the USA and Europe could not be transferable to other countries due to variability in the structure and delivery of healthcare systems in each country. Studies in other countries/world regions are therefore needed.

Next, only studies comparing operative and non-operative treatment were included in this review. Therefore, studies that evaluated only one of the two treatment options were excluded, and this might represent a bias. Due to variability in the delivery of health service, specificity in cost, and the diversity of usual care in each region, we chose only comparative studies instead of studies evaluating cost analysis for each treatment approach for comparing directly between ACLR versus non-operative treatment. Although the total number of included studies was quite low, all had an appropriate score on the CHEC list, so the findings of this systematic review can be considered low in risk of bias and provide valuable information for supporting clinical guidelines in this field. This study showed the most cost-effective treatment for each individual patient as soon as the diagnosis of ACL injury has been made. Clinicians can use the findings of this review in their day-to-day clinical work by including the age and activity level of each patient in the decision-making process after an ACL injury.

## Conclusion

Early ACLR is likely a more cost-effective strategy for ACL injury in athletes and young people with high-activity levels. By contrast, non-operative treatment with the optional delayed ACLR may be a more cost-effective strategy in a middle-aged population with moderate activity levels. As the total number of studies included in this review was low, more studies are needed to enhance decision-making in clinical practice regarding the best possible as well as the most cost-effective treatment for ACL injury for each individual.

## Supplementary Information

Below is the link to the electronic supplementary material.

Supplementary file1 (DOCX 17 KB)
