# Correction to: “Cost-effectiveness of ACL treatment is dependent on age and activity level: a systematic review”

**DOI:** 10.1007/s00167-022-07143-8

**Published:** 2022-09-14

**Authors:** R. Deviandri, H. C. van der Veen, A. M. T. Lubis, I. van den Akker-Scheek, M. J. Postma

**Affiliations:** 1 Department of Orthopedics, University of Groningen, University Medical Center Groningen, Hanzeplein 1, 9713 GZ Groningen, The Netherlands; 2 Department of Physiology, Faculty of Medicine, Universitas Riau, Pekanbaru, Indonesia; 3 Division of Orthopedics, Arifin Achmad Hospital, Pekanbaru, Indonesia; 4 Department of Orthopedics-Faculty of Medicine, Universitas Indonesia/Cipto Mangunkusumo Hospital, Jakarta, Indonesia; 5 Department of Health Sciences, University of Groningen, University Medical Center Groningen, Groningen, The Netherlands; 6 Department of Economics, Econometrics & Finance, Faculty of Economics & Business, University of Groningen, Groningen, The Netherlands; 7 Department of Pharmacology & Therapy, Universitas Airlangga, Surabaya, Indonesia; 8 Center of Excellence in Higher Education for Pharmaceutical Care Innovation, Universitas Padjadjaran, Bandung, Indonesia

## Correction to: Knee Surgery, Sports Traumatology, Arthroscopy 10.1007/s00167-022-07087-z

Authors would like to update the missing text in the abstract section.

In the published version:

On the other hand, non-operative treatment with optional delayed ACLR may be the more cost-effective.

However, it should be:

On the other hand, non-operative treatment with optional delayed ACLR may be the more cost-effective strategy in the middle age population with moderate activity levels.

The original article has been corrected.

