# Supplementary material for: “Cost-effectiveness of ACL treatment is dependent on age and activity level: a systematic review”
Source: Knee Surg Sports Traumatol Arthrosc. 2022 Aug 23;31(2):530–41. doi: 10.1007/s00167-022-07087-z (PMC9898360; doi:10.1007/s00167-022-07087-z)
Supplement: Supplementary file 1 — Supplementary file1 (DOCX 17 KB) [file 167_2022_7087_MOESM1_ESM.docx]

|  | **CHEC-list** | Gottlob (11] | Farshad [13] | Mather [18] | Mather [19] | Kiadaliri [16] | Stewart [32] | Eggerding [7] |  |
| --- | --- | --- | --- | --- | --- | --- | --- | --- | --- |
| 1 | Was the study population clearly described? | Y | Y | Y | Y | Y | Y | Y |  |
| 2 | Were competing alternatives clearly described? | Y | Y | Y | Y | Y | Y | Y |  |
| 3 | Was a well-defined research question posed in answerable form? | Y | Y | Y | Y | Y | Y | Y |  |
| 4 | Was the economic study design appropriate to the stated objective? | Y | Y | Y | Y | Y | Y | Y |  |
| 5 | Was the chosen time horizon appropriate in order to include relevant costs and consequences? | N | N | Y | N | N | N | N |  |
| 6 | Was the actual perspective chosen appropriate? | N | N | Y | Y | Y | Y | Y |  |
| 7 | Were all important and relevant costs for each alternative identified? | Y | Y | Y | Y | Y | Y | Y |  |
| 8 | Were all costs measured appropriately in physical units? | Y | Y | Y | Y | Y | Y | Y |  |
| 9 | Were costs valued appropriately? | Y | Y | Y | Y | Y | Y | Y |  |
| 10 | Were all important and relevant outcomes for each alternative identified? | N | Y | N | Y | Y | Y | Y |  |
| 11 | Were all outcomes measured appropriately? | Y | Y | Y | Y | Y | Y | Y |  |
| 12 | Were outcomes valued appropriately? | Y | Y | Y | Y | Y | Y | Y |  |
| 13 | Was an incremental analysis of costs and outcomes of alternatives performed? | Y | Y | Y | Y | Y | Y | Y |  |
| 14 | Were all future costs and outcomes discounted appropriately? | Y | N | Y | Y | Y | Y | Y |  |
| 15 | Were all important variables whose values are uncertain appropriately subjected to sensitivity analysis? | Y | Y | Y | Y | Y | Y | Y |  |
| 16 | Did the conclusions follow from the data reported? | Y | Y | Y | Y | Y | Y | Y |  |
| 17 | Did the study discuss the generalizability of the results to other settings and patient/client groups? | Y | Y | Y | Y | Y | Y | Y |  |
| 18 | Did the article indicate that there is no potential conflict of interest between study researcher(s) and funder(s)? | N | Y | Y | Y | Y | Y | Y |  |
| 19 | Were ethical and distributional issues discussed appropriately? | Y | Y | Y | Y | Y | Y | Y |  |
| **Total score** | | **15** | **16** | **18** | **18** | **18** | **18** | **18** |  |

**Appendix. Supplementary data**

**Table A.1 Quality assessment of each study according to CHEC list**
